# Impact of Warming on Greenhouse Gas Production and Microbial Diversity in Anoxic Peat From a *Sphagnum*-Dominated Bog (Grand Rapids, Minnesota, United States)

**DOI:** 10.3389/fmicb.2019.00870

**Published:** 2019-04-26

**Authors:** Max Kolton, Ansley Marks, Rachel M. Wilson, Jeffrey P. Chanton, Joel E. Kostka

**Affiliations:** ^1^School of Biology, Georgia Institute of Technology, Atlanta, GA, United States; ^2^Department of Earth, Ocean & Atmospheric Science, Florida State University, Tallahassee, FL, United States; ^3^School of Earth and Atmospheric Science, Georgia Institute of Technology, Atlanta, GA, United States

**Keywords:** peatlands, climate change, methanogenesis, microbial community, microbial diversity

## Abstract

Climate warming is predicted to increase heterotrophic metabolism in northern peatland soils leading to enhanced greenhouse gas emissions. However, the specific relationships between temperature and the greenhouse gas producing microbial communities are poorly understood. Thus, in this study, the temperature dependence of carbon dioxide (CO_2_) and methane (CH_4_) production rates along with abundance and composition of microbial communities were investigated in peat from a *Sphagnum*-dominated peatland, S1 bog (Minnesota, United States). Whereas CH_4_ production rates increased with temperature up to 30°C, CO_2_ production did not, resulting in a lower CO_2_:CH_4_ ratio with increasing temperature. CO_2_ production showed both psychrophilic and mesophilic maxima at 4 and 20°C, respectively, and appears to be mediated by two anaerobic microbial communities, one that operates under psychrophilic conditions that predominate for much of the year, and another that is more active under warmer conditions during the growing season. In incubations at 10°C above the ambient range, members of the *Clostridiaceae* and hydrogenotrophic methanogens of the *Methanobacteriaceae* dominated. Moreover, a significant negative correlation between temperature and microbial diversity was observed. Results indicate that the potential consequences of warming surface peat in northern peatlands include a large stimulation in CH_4_ production and a significant loss of microbial diversity.

## Introduction

Covering approximately 3% of the land surface while storing up to one-third of global soil carbon, boreal peatlands are effective carbon sinks. At the same time, boreal peatlands represent one of the main natural sources of the greenhouse gas methane (CH_4_) (Gorham, 1991; Tarnocai et al., 2009; Dlugokencky et al., 2011). Climate models predict an increasing contribution of these ecosystems to climate change due to an estimated 6–10°C temperature elevation by the end of the 21st century that will likely stimulate microbial activities, CO_2_ and CH_4_ production (Collins et al., 2014; Wu and Roulet, 2014; Schuur et al., 2015; Schadel et al., 2016). However, most existing climate models lack detailed input parameters specifically defining the temperature effect on microbial community composition and activity.

Boreal peatland soils are saturated with water and anoxic conditions prevail belowground (Limpens et al., 2008). Anaerobic decomposition of organic matter in peat is mediated by a complex web of metabolic interactions between many microbial groups (Drake et al., 2009). Plant-derived organic matter (cellulose, hemicellulose, pectin) is first hydrolyzed into sugars, fermented to organic acids (acetate, propionate, butyrate, etc.) or alcohols, and then terminally metabolized to CO_2_ and CH_4_. Since the majority of peatlands harbor low concentrations of inorganic electron acceptors such as nitrate (${\mathrm{NO}}_{3}^{–}$), manganese (Mn [IV]), ferric iron (Fe [III]), or sulfate (${\mathrm{SO}}_{4}^{2–}$), methanogenesis is considered to be the predominant terminal electron accepting process in peatland soils (Conrad, 2009; Bridgham et al., 2013). Methanogenesis is an anaerobic process carried out by archaea, mainly those taxonomically affiliated with the *Euryarchaeota* group; however, methane production has been observed in aerobic soils (Angle et al., 2017). In addition, some saprotrophic fungi, taxonomically affiliated with the *Basidiomycetes*, and bacteria affiliated with *Cyanobacteria* are able to produce CH_4_ under oxic conditions (Lenhart et al., 2012; Bižić-Ionescu et al., 2018). These recent findings challenge the dogma that methanogenesis is exclusive to strictly anaerobic Archaea. Under anoxic conditions, methanogenic archaea produce CH_4_ through three major pathways (1) from CO_2_ and H_2_ (hydrogenotrophic pathway), (2) acetate disproportionation (acetoclastic pathway), and (3) cleavage of methylated compounds (Whitman et al., 2006; Thauer et al., 2008; Borrel et al., 2013, 2016; Offre et al., 2013). Peat temperature, water table depth, and microbial community composition are among the major factors that control methanogenesis (Dunfield et al., 1993; Schulz and Conrad, 1996; Metje and Frenzel, 2005, 2007; Tveit et al., 2015; Peltoniemi et al., 2016; Wilson et al., 2016; Zalman et al., 2018). While peat temperature and the availability of thermodynamically preferable terminal electron acceptors have a direct effect on methanogenesis, the microbial community response to temperature may have broader ecosystem consequences. Nevertheless, the response of peat microbial communities to rising temperatures is poorly understood.

The consensus of previous work is that climate warming is likely to result in an acceleration of organic matter decomposition, and stimulation of CO_2_ and CH_4_ emissions in peatlands (Davidson and Janssens, 2006; Weedon et al., 2013). Since CH_4_ has a sustained global warming potential of 34-times that of CO_2_ over a 100-year time frame, enhanced CH_4_ emission is likely to result in a positive feedback to climate warming (Myhre et al., 2014; Neubauer and Megonigal, 2015). Thus, understanding the temperature dependence of microbially-mediated organic matter turnover and CH_4_ production is critical for predicting ecosystem responses to climate change drivers (McGuire and Treseder, 2010; Wallenstein and Hall, 2012; Schimel, 2013; Wieder et al., 2013; Ma et al., 2017).

The objective of this study was to quantify the temperature response of anaerobic organic matter decomposition to CO_2_ and CH_4_ in surficial soils from a *Sphagnum*-dominated peatland. Moreover, we aimed to characterize the abundance and composition of microbial communities that developed across the range of temperature conditions. Environmental warming resulted in a significant microbial diversity loss. Additionally, a strong positive correlation was observed between temperature and CH_4_ production, leading to a large decrease in the CO_2_:CH_4_ ratio.

## Materials and Methods

### Sample Collection

Peat samples were collected from the S1 Bog, located within the Marcell Experimental Forest (U.S. Forest Service), approximately 40 km north of Grand Rapids, Minnesota, United States (47°30.476′ N; 93°27.162′ W; 418 m above mean sea level). The S1 Bog is a part of the SPRUCE (Spruce and Peatland Responses Under Changing Environments) experiment which investigates the effect of whole ecosystem warming and elevated CO_2_ concentrations on peatland ecosystem processes since June 2014 (Wilson et al., 2016; Hanson et al., 2017). The S1 Bog is characterized as an ombrotrophic, nutrient-deficient, acidic, *Sphagnum*-dominated peat bog (surface pH < 4.0) with undetectable levels of dissolved O_2_ (∼20 ppb) below the top 5 cm (Lin et al., 2014a; Warren et al., 2017). S1 bog receives water and nutrient inputs primarily from precipitation. Annual precipitation and air temperature average 768 mm and 3.3°C respectively (Sebestyen et al., 2011). Samples were obtained from a methanogenic layer at 10–30 cm below surface (Lin et al., 2014b; Tfaily et al., 2014; Wilson et al., 2016) in June 2013. The methanogenic layer was homogenized in a sterile bag. Approximately 5 g of the homogenate were immediately frozen on dry ice for microbiological community analysis.

### Temperature Gradient Block Experiments

Peat samples were diluted 1:1 (vol/vol) with O_2_-free autoclaved distilled water and homogenized under anaerobic conditions. The resulting slurries (∼10 g) were placed into sterile test tubes (30 ml). Tubes were closed with butyl rubber stoppers, capped, and flushed with nitrogen gas (N_2_) for 5 min. Prior to the experiment, the tubes were pre-incubated for 10 days in a custom-made temperature gradient block to allow the system to equilibrate. During the pre-incubation and experimental time courses, the temperature gradient block was set to a stable gradient of 0 to 40°C at temperature intervals of approximately 2–3°C (Canion et al., 2014). After 10 days of pre-incubation, the tubes were flushed with N_2_ for 5 min. Three replicates per temperature were incubated for 4 weeks in the temperature gradient block. The headspace concentrations of CH_4_ and CO_2_ were measured periodically, usually a few times a week, after shaking to equilibrate the gas and liquid phases. At the end of the incubation period, peat from incubation tubes was frozen and stored at -80°C for DNA extraction and molecular analysis.

### Quantification of CO_2_ and CH_4_

Gas headspace concentrations were monitored as previously described (Esson et al., 2016). Briefly, headspace gas samples (25–200 μl) were injected into a Shimadzu GC-2014 with a Supelco custom-packed column (Packing 80/100 Hayesep Q) coupled to a flame ionization detector (FID) equipped with a methanizer. The flow rate was 30 ml/min with the injector and detector at 100°C, the column at 40°C, and the methanizer at 380°C. The sampled gas was not replaced due to the relatively short incubation and minimal headspace sampling. Moles of gas were calculated based on standard calibration curves and adjusted according to Henry’s law of gas solubility in water. Finally, total generated gas was normalized to the dry weight of peat added to each bottle (approximately 3–5% of the slurry wet weight). Maximum potential production rates and accumulation of gasses were calculated from a growth model fitted by splines with the R package “grofit” (Kahm et al., 2010).

### Microbial Community Analyses

Duplicate samples were randomly selected from triplicate peat microcosms for extraction of environmental DNA at the end of the incubation experiment using the MoBio PowerSoil DNA extraction kit according to the manufacturer’s protocol (MoBio Laboratories, Carlsbad, CA, United States). From each duplicate, two technical replicates were extracted, resulting in four DNA pools for each of the temperature treatments. Extracted DNA was quantified with the Qubit HS assay kit (Invitrogen, Carlsbad, CA, United States) and 10 ng per reaction was used to generate SSU rRNA amplicons. Prokaryotic community composition was determined by applying a high-throughput sequencing-based protocol that targets PCR-generated amplicons from the V4 variable regions of the SSU rRNA gene using the primer set CS1_515F (5′-ACACTGACGACATGGTTCTACA_GTGCCAGCMGCCGCGGTAA) and CS2_806R (5′-TACGGTAGCAGAGACTTGGTCT_GGACTACHVGGGTWTCTAAT) (Caporaso et al., 2010; Moonsamy et al., 2013). Resulting SSU rRNA gene amplicons were barcoded with unique 10-base barcodes (Fluidigm Corporation, South San Francisco, CA, United States) and sequenced on an Illumina MiSeq2000 platform at the Georgia Institute of Technology as previously described (Wilson et al., 2016; Carrell et al., 2019). The Illumina-generated SSU rRNA gene amplicon sequences have been deposited in the BioProject database^1^ under accession PRJNA431624.

### Sequence Processing and Analysis

Initially, Illumina-generated SSU rRNA gene sequences from raw fastq files were screened for matching forward and reverse amplification primers, using Cutadapt v.1.8.1 (Martin, 2011). The resulted sequences were quality-filtered and assembled into error-corrected amplicon sequence variants (ASVs) using DADA2 v1.8.0 (Callahan et al., 2016), which represent unique prokaryotic taxa. Unique ASVs were aligned in Mothur v.1.39.0 software (Schloss et al., 2009) using SILVA reference database (Quast et al., 2013). An approximately maximum-likelihood tree was constructed from the alignment of ASV’s using FastTree v.2.1.1 (Price et al., 2010). Taxonomic assignments of these high-quality sequences were annotated to the SILVA 132 database using the RDP classifier (Wang et al., 2007) with a minimum confidence threshold of 50%. Sequences classified as “chloroplast,” “mitochondria” or did not match any taxonomic “Class” were removed from the final dataset. The ASV-based alpha-diversity indexes were calculated based on the number of unique ASVs (richness) and Faith’s phylogenetic diversity (PD) (Faith, 1992) to assess phylogenetic alpha-diversity. Estimates of alpha diversity indexes were performed based on randomly selected 1100 microbial sequences per sample. Prior to microbial composition and beta-diversity analysis, the final dataset was normalized by cumulative sum scaling (CSS) (Paulson et al., 2013). To quantify the major variance components of beta-diversity the unconstrained principal coordinates analysis (PCoA) based on Bray–Curtis, weighted, and unweighted unifrac (Lozupone and Knight, 2005) dissimilarities was performed. The temperature effect on community dissimilarity was estimated with a permutational analysis of variance (PERMANOVA) and permutational analysis of multivariate dispersions (BETADISP) with 1000 permutations. Statistical analyses were conducted in R v3.5 using phyloseq and vegan R packages (McMurdie and Holmes, 2013; Oksanen et al., 2015; R Development Core Team, 2018).

### Quantitative PCR Amplification

All quantitative polymerase chain reaction assays were performed in triplicate using the StepOnePlus platform (Applied Biosystems, Foster City, CA, United States) and PowerUp SYBR Green Master Mix (Applied Biosystems, Foster City, CA, United States). Universal bacterial and archaeal gene copy number was enumerated based on small subunit rRNA genes (SSU rRNA). Quantification of bacterial and archaeal SSU rRNA gene copies was carried out in a final volume of 20 μl using standard primer sets 331F (5′-CCTACGGGAGGCAGCAGT)/518R (5′-ATTACCGCGGCTGCTG) and Arch787F (5′-ATTAGATACCCSBGTAGTCC)/Arch1059R (5′-GCCATGCACCWCCTC) respectively (Muyzer et al., 1993; Yu et al., 2005) and previously described conditions (Warren et al., 2017; Carrell et al., 2019). Standard curves were obtained with a 10-fold serial dilution of standard pGEM-T Easy plasmids (Promega, Madison, WI, United States) containing target sequences from *Escherichia coli* K12 or *Methanococcus maripaludis S2* for bacteria and archaea, respectively. Standard curves ranged from 10^2^ to 10^7^ molecules, and negative controls containing no template DNA to exclude or detect any possible contamination were run simultaneously on each PCR plate. Specificity of PCR products was confirmed by melting curve analyses and gel electrophoresis of the amplicons. Bacterial and archaeal 16S rRNA gene copy numbers were calculated and presented as gene copy numbers per dry gram of peat.

## Results

Surface peats from a northern Minnesota bog were incubated anaerobically over a 4-week period in the temperature range from 0 to 40°C, and gas production was monitored. Following the incubation, microbial community composition was determined using next generation sequencing of SSU rRNA gene amplicons and microbial abundance was quantified by qPCR with domain level primers.

### Temperature Response of Greenhouse Gas (GHG) Production Under Anoxic Conditions

**FIGURE 1:**
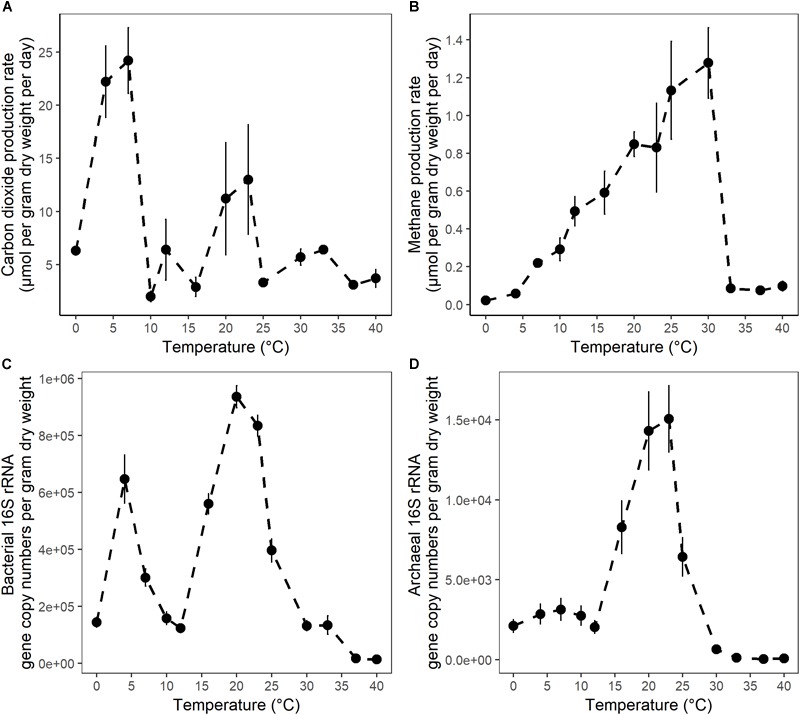
The temperature dependence of **(A)** CO_2_ maximum potential production rates; **(B)** CH_4_ maximum potential production rates; **(C)** bacterial 16S rRNA gene copy numbers; and **(D)** archaeal 16S rRNA gene copy numbers in anaerobic incubations of surface peat collected from the S1 bog in northern Minnesota. Microbial 16S rRNA gene copy numbers were determined in samples collected after 4 weeks of incubation by qPCR with bacterial or archaeal primers. The error bars show the standard deviation for each temperature treatments (*n* = 3).

**FIGURE 2:**
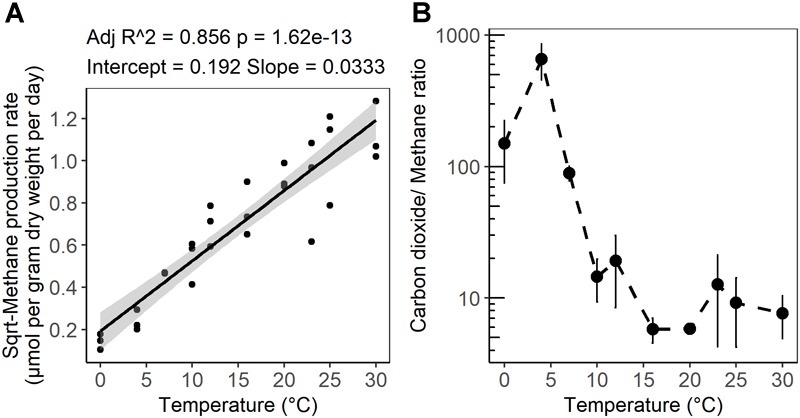
**(A)** Linear regression of the square root of the CH_4_ production rate as a function of incubation temperature. **(B)** Temperature dependence of the ratio of CO_2_ produced to CH_4_ produced after 4 weeks of anaerobic incubation at different temperatures. The shaded area shows 95% confidence intervals. The error bars show the standard deviation for each temperature treatments (*n* = 3).

After 10 days of pre-incubation, maximum potential rates of GHG production were determined during 4 weeks of anoxic incubation at 13 incubation temperatures ranging from 0 to 40°C at 2–3°C intervals. CO_2_ and CH_4_ maximum potential rates had contrasting responses to temperature (Figure 1A,B). Production of CO_2_ was observed after 1 day at all incubation temperatures and reached a maximum potential rate after 0.04 ± 0.1 to 6.8 ± 0.6 days in 33 and 4°C treatments, respectively (Supplementary Figure S1A and Table S1). The time elapsed from the start of the incubation to the onset of maximum production rates, referred to as “lag time,” varied with temperature (Supplementary Table S1). Average lag times for CO_2_ production were 9.72 ± 3 and 2.4 ± 2.6 days for temperature treatments below and above 12°C, respectively (Supplementary Table S1). Methane production also varied with temperature. For temperature treatments between 16 and 30°C, accumulation of CH_4_ was observed after 1 day of incubation, while a 4.1 ± 2 and 7 ± 2.8 days lag time was observed in 10 and 7°C temperature treatments, respectively. Lag times for CH_4_ production were 7 ± 2.5 and 1 ± 1.8 days for temperatures below 12°C and between 12 and 30°C, respectively (Supplementary Table S1). Two major temperature optima for CO_2_ maximum potential rates were observed, at 4–7 and 20–23°C, with maximum potential rates of 23.2 ± 1.4 and 12.1 ± 1.2 μmol per gram dry weight per day, respectively (Figure 1A and Supplementary Table S1). Methane maximum potential rates ranged from 0.02 ± 0.01 to 1.28 ± 0.2 μmol per gram dry weight per day across temperature treatments (Figure 1B, 2A and Supplementary Table S1). While maximum potential rates of methanogenesis exponentially increased between 0 and 30°C, reaching a maximum potential rate of 1.28 ± 0.2 μmol per gram dry weight per day at 30°C, no or limited CH_4_ production was observed below 4°C and above 30°C (Figure 1B, 2A and Supplementary Table S1). The ratio of accumulated CO_2_ and CH_4_, as calculated at the end of incubation, showed a temperature-dependent decline from 656.5 ± 200 at 4°C down to 5.8 ± 0.3 at 20°C respectively (Figure 2B and Supplementary Table S1).

### Quantification of Microbial Abundance

To quantify the effect of incubation temperature on the abundance of microbial communities, quantitative PCR (qPCR) analysis was performed. Results are reported as the abundance of SSU rRNA gene copy numbers per gram dry weight of peat. Bacterial 16S rRNA gene copy numbers ranged over nearly two orders of magnitude from 1.45 × 10^4^± 1.2 × 10^3^ to 9.36 × 10^5^± 3.9 × 10^4^ at 40 and 20°C respectively. Additionally, two maxima in gene copy numbers were observed at 20–23 and 4°C (Figure 1C and Supplementary Table S2), suggesting that bacterial abundance varied with temperature. Moreover, these maxima in microbial gene copy numbers correlated well with observed maxima in potential CO_2_ production rates (Figure 1A). In contrast, archaeal 16S rRNA gene copy numbers ranged over nearly three orders of magnitude from 3.42 × 10^1^± 2.6 × 10^1^ to 1.51 × 10^4^± 2.1 × 10^3^ and were characterized by a single maximum at 20–23°C (Figure 1D and Supplementary Table S2). Archaeal 16S rRNA gene copy numbers were highly correlated with maximum potential rates of CH_4_ production (Figure 1B). Finally, less than 100 archaeal 16S rRNA gene copies were found at temperatures where no or limited CH_4_ production was detected (Figure 1B,D and Supplementary Figure S1, Supplementary Tables S1, S2).

### Microbial Community Dynamics With Temperature

**FIGURE 3:**
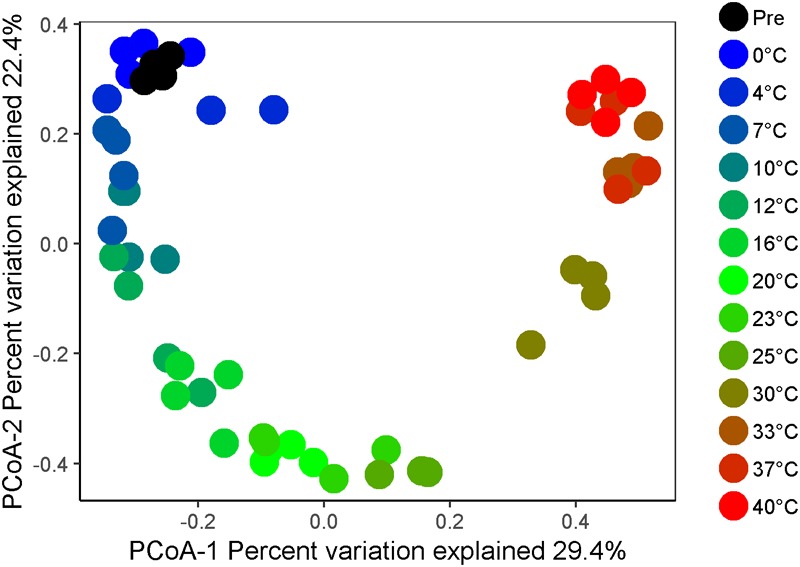
Beta diversity of microbial communities as a function of temperature after 4 weeks of anaerobic incubation. The plot represents a principal coordinates analysis (PCoA) of microbial community compositions based on Bray–Curtis distance matrices calculated after cumulative sum scaling (CSS) normalization of the final data.

**FIGURE 4:**
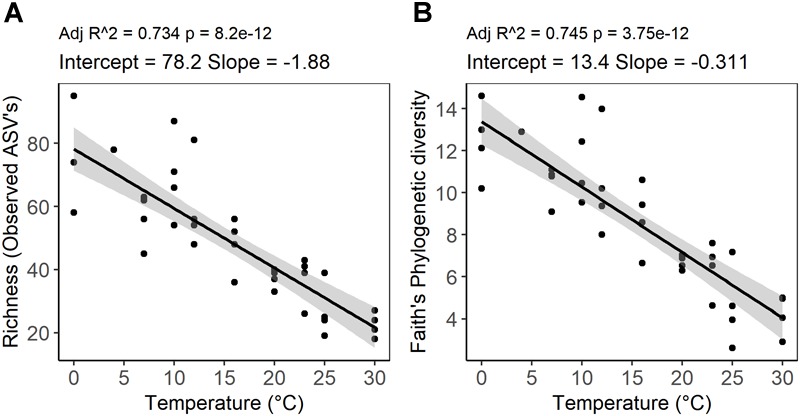
Temperature dependence of alpha diversity of peat microbial communities after 4 weeks of anaerobic incubation determined by **(A)** richness as an abundance of ASVs and **(B)** Faith’s phylogenetic diversity (PD). The graph represents a linear regression between alpha diversity indices and incubation temperature. The shaded area shows 95% confidence intervals.

**FIGURE 5:**
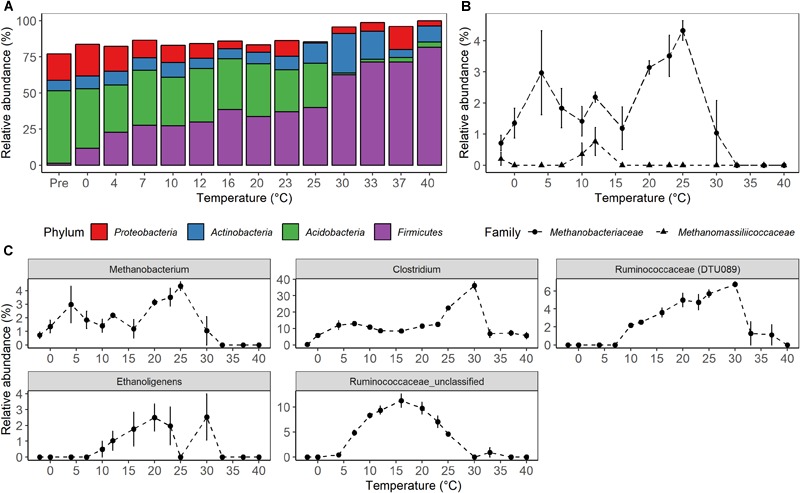
Microbial taxonomic composition as a function of temperature after 4 weeks of anaerobic incubation. **(A)** Relative abundance of the most abundant bacterial phyla. **(B)** Relative abundance of known groups of methanogens within the *Euryarchaeota*, and **(C)** relative abundance of genera affiliated with *Clostridiales* having similar abundance profile with methane production rates. The relative abundance of the sequences assigned to a given taxonomic level was calculated for each of the biological replicate, and the average value was then used to represent the relative abundance of each temperature treatment. The treatment “Pre” on the **(A)** and “-2” on the **(B,C)** is a microbial community of the unincubated peat samples. The error bars show the standard deviation of relative abundance for each temperature treatments (*n* = 4).

High-throughput sequencing of small subunit ribosomal RNA genes yielded a total of 195,418 high-quality sequences (range 1,100–8,448; median 3,530 sequences per sample), and revealed profound changes in microbial community diversity and composition with temperature (Figure 3–5). The Bray–Curtis based PCoA and permutational multivariate analysis of variance (PERMANOVA) of beta-diversity clearly showed variations in microbial communities between temperature treatments (*R^2^* = 0.75, *P* < 0.001, Figure 3 and Supplementary Table S3). Elevated temperature resulted in a significant reduction in microbial community richness and PD (Figure 4). Community richness, determined as observed ASV’s and Faith’s PD, decreased up to threefold in a temperature dependent manner by the end of the incubation period (Supplementary Table S4). The observed relative low alpha diversity indices may be due to limited sequencing depth, nevertheless, the observed pattern of decreasing diversity with increasing temperatures will not likely change as a result of higher sequencing efforts. Taxonomic community composition varied with temperature in parallel with changes in diversity. The relative abundance of members of the *Acidobacteria* phylum decreased during the incubation period in a temperature dependent manner. The temperature effect on *Acidobacteria* was stronger at higher compared to lower incubation temperatures. While at the end of the incubation the relative abundance of *Acidobacteria* was 30–40% in the warming treatments between 0 and 25°C, their relative abundance at 30°C was only 1.3% (Figure 5A and Supplementary Table S5). The *Firmicutes* comprised 1.3 ± 0.4% of the total microbial community in the unincubated peat samples; whereas, their relative abundance increased in a temperature dependent manner by the end of incubation. The relative abundance of *Firmicutes* at the end of incubation was 11.7 ± 0.7% at 0°C compared to 81.6 ± 5.6% at 40°C (Figure 5A and Supplementary Table S5). Microbial communities established at the thermal optimum of 4–7°C were significantly different from communities established at the 20–23°C optimum (Supplementary Table S3). Substantial differences were observed in the relative abundances of the *Alphaproteobacteria* and *Acidobacteriia* classes in the temperature range relevant to field conditions. Members of the *Alphaproteobacteria* were more susceptible to temperature changes than members of the *Acidobacteriia*. The relative abundance of *Alphaproteobacteria* at the end of the incubation period was 11.7 ± 1.45% at 4°C compared to 2.4 ± 0.8% at 20°C (Supplementary Table S6). In contrast, the relative abundance of *Acidobacteriia* was 25.9 ± 5% at 4°C and 35.9 ± 2.9% at 20°C at the end of the incubation (Supplementary Table S6). The dominant *Firmicutes* family in the peat incubation was *Clostridiaceae* with a relative abundance at 30°C of 41.2 ± 2.3% (Supplementary Table S7). The relative abundance of the archaeal phylum *Euryarchaeota* that hosts the majority of known methanogens correlated well with CH_4_ production. *Euryarchaeota* relative abundance was fairly stable at temperatures below 12°C, while abundance showed a larger increase in temperature treatments above 12°C, reaching a maximum relative abundance of 4.3 ± 0.3% at 25°C (Supplementary Table S5). Hydrogenotrophic methanogens taxonomically affiliated with the *Methanobacteriaceae* were dominant members of the methanogenic communities that developed with increasing temperature. Members of the *Methanobacteriaceae* family comprised over 90% of methanogenic communities at all temperatures (Figure 5B). The relative abundance of the *Methanobacterium* genus correlated well with maximum CH_4_ production rates (Figure 1B) and the abundance of the hydrogen-producing genera within *Clostridiales* order (Figure 5C). Additionally, we were unable to detect the methanogens in communities developed at 40°C (Figure 5B and Supplementary Table S7), where an undetectable level of CH_4_ production and less than 100 copies of archaeal SSU rRNA genes were detected (Figure 1B,D and Supplementary Table S2).

## Discussion

Microorganisms play a key role in greenhouse gas exchange at the soil-atmosphere interface in northern peatlands by mediating the decomposition of organic matter to CO_2_ and CH_4_. The majority of previous studies predict that CH_4_ and CO_2_ emission rates should increase as a result of warming (Yvon-Durocher et al., 2012, 2014; Wu and Roulet, 2014; Schuur et al., 2015; Schadel et al., 2016; Wilson et al., 2016). While the response of CH_4_ production to temperature is well-studied in wetlands including peatlands, *Sphagnum*-dominated peatlands emit a high proportion of CO_2_ in comparison to CH_4_, and surprisingly little information is available on the temperature dependence of anaerobic CO_2_ production in wetlands (Tfaily et al., 2014; Treat et al., 2015; Tveit et al., 2015; Schadel et al., 2016; Wilson et al., 2016). In addition, few previous studies have linked the temperature response of greenhouse gas production to microbial community dynamics. The present study provides a quantification of the temperature response of microbial community dynamics and resulting greenhouse gas production in surface peat from a *Sphagnum*-dominated peatland, the S1 bog, in northern Minnesota.

### Temperature Response of CH_4_ and CO_2_ Production

Consistent with previous observations from laboratory and field studies of wetland soils including peatlands, methane production rates from the subsurface of S1 bog were positively correlated with temperature and a mesophilic optimum was observed at 30°C. In agreement with field observations (Wilson et al., 2016) an exponential relationship between CH_4_ production and temperature elevation was observed under laboratory conditions (Figure 1B). Nevertheless, other autotrophic and heterotrophic processes such as methane oxidation, photosynthesis, and plant respiration are omitted from *in vitro* observations, and these may have differential sensitivity to temperature elevation. Regression analysis of CH_4_ maximum potential rates indicated that temperature changes between 0 and 30°C accounted for up to 86% of the variation in CH_4_ maximum potential rates (Figure 2A). These variations were correlated with archaeal community size and relative abundance. The mesophilic response of methanogens can be explained by the fact that the S1 bog represents a temperate peatland (Tveit et al., 2015). In this study, as well as in studies from other temperate soils, the CH_4_ maximum potential rates at 4°C comprised only 1 to 10% of the rates determined at 25 to 30°C (Glissman et al., 2004; Freitag and Prosser, 2009). However, the CH_4_ maximum potential rates at 7°C was approximately 20% of rates observed at 25 and 30°C. In higher latitude Arctic or subarctic soils, CH_4_ maximum potential rates at 4°C were approximately 17 to 25% off maximum potential rates determined between 25 and 30°C respectively (Metje and Frenzel, 2007; Tveit et al., 2015). These observations may suggest the existence of latitude-dependent eco-physiological adaptation of the methanogenic communities to survive under psychrophilic conditions. In a global meta-analysis of methanogenesis rates obtained from field and laboratory studies of soils in wide-ranging ecosystems (Yvon-Durocher et al., 2014) the average temperature dependence corresponded to a 57-fold increase between 0 and 30°C. Our results corroborate previous work as we observed an approximately 60-fold increase in methanogenesis maximum potential rates across the same range in temperature.

In contrast to methanogenesis, few studies have examined the temperature response of organic matter decomposition to CO_2_ in wetlands or other terrestrial soils under anaerobic conditions. In agreement with previous global compilations of soil respiration rate measurements (Yvon-Durocher et al., 2012; Schadel et al., 2016), in our study CO_2_ maximum potential rates, a proxy for respiration, did not show as large of a temperature dependence as methanogenesis, resulting in decreasing CO_2_:CH_4_ ratios with warming. To our knowledge, this study is among the first in peatland soils to observe that CO_2_ maximum potential rates are highest in the psychrophilic range. However, the observed CO_2_ maximum potential rates may be at least partially due to faster utilization of the available electron acceptors pools under warmer condition during the pre-incubation period. The majority of previous studies in soils have observed a continuous increase in CO_2_ production over the temperature range studied, from 0 to 40 or 60°C, although the majority of previous studies included relatively few temperature treatments (Metje and Frenzel, 2005, 2007; Pietikåinen et al., 2005; Hoj et al., 2008; Tveit et al., 2015). However, similar to our results, Metje and Frenzel (2005) observed comparable dynamics of respiration rates at 0 and 25°C, in subsurface soils from a peatland in northern Finland.

In further corroboration of our results, similar temperature response trends were observed for rates of anaerobic microbial respiration processes linked to organic matter degradation in temperate wetlands. For example, the highest denitrification rates were observed under psychrophilic conditions in saltmarsh sediments collected in winter (Kaplan et al., 1977). Moreover, in another study of saltmarsh sediments, two separate rate maxima were observed in the temperature response of denitrification rates. The temperature of the psychrophilic optimum varied with season, whereas the mesophilic optimum did not (King and Nedwell, 1984). In the present study, two separate CO_2_ production maxima were observed in the psychrophilic (4–7°C) and mesophilic temperature ranges (20–23°C), respectively (Figure 1A), suggesting that distinct microbial communities are present in surface peat that are active in these different temperature ranges.

Maxima in CO_2_ production occurred at ranges that match with *in situ* temperatures measured in winter and summer seasons in the S1 bog. Surficial S1 bog soils represent a cold temperate environment, during the winter season (November–April), with monthly average temperatures in the top 30 cm depth ranging from -0.7 to 0.5°C. During the summer season (June–September), monthly average temperatures in the 0 to 30 cm depth interval range from 12.9 to 17.1°C (with a maximum observed at 21.5°C). Thus, maxima in CO_2_ maximum potential rates observed at 4 to 7°C as well as 20 to 23°C indicate that one community is adapted to *in situ* psychrophilic conditions in winter while another becomes active at moderate temperatures during the period of plant growth. CO_2_ maximum potential rates measured under psychrophilic conditions were higher by approximately a factor of 2, and abundance of bacteria, not archaea, closely paralleled with the CO_2_ production rate. Although gas production was not investigated during the pre-incubation period, extensive analysis of gas accumulation and of *in situ* microbial abundance shows that bacteria are 1 to 2 orders of magnitude more abundant than archaea in surface peats of the S1 bog (Lin et al., 2014a; Wilson et al., 2016). Since organic matter is mostly decomposed to CO_2_ in the S1 bog, our results indicate that heterotrophic bacteria, and not methanogenic archaea, are responsible for the majority of organic matter mineralization under *in situ* conditions.

Further research is needed to determine the mechanisms controlling the temperature dependence of anaerobic respiration in wetland soils. Studies conducted with field samples in the laboratory often interrogate different temperature ranges or incubation times and calculate respiration rates using different protocols that impact interpretation of the temperature response. Overall, uncertainties in the temperature response of anaerobic CO_2_ production due to respiration in soils may be due to soil heterogeneity, a paucity of CO_2_ production or respiration measurements, and a lack of standard protocols for quantifying and compiling rates. Hence, as has been done for methanogenesis, rates of anaerobic CO_2_ production should be quantified using standard protocols in soils from a wide range of ecosystems.

In *Sphagnum*-dominated peatlands such as the S1 bog, the soils are generally devoid of inorganic electron acceptors below the top few centimeters and methanogenesis is considered to be the predominant terminal electron accepting process (Lin et al., 2014b; Tfaily et al., 2014). Methanogenesis is predicted to produce a stoichiometric ratio of 1:1 CO_2_:CH_4_, [(CH_2_O)_n_ 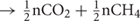; (Conrad, 1999)]. However, as has been observed previously in peatlands (Wilson et al., 2017), higher CO_2_:CH_4_ ratios were observed in this study, and the ratio showed a temperature-dependent decline from 656.5 ± 200 at 4°C down to 5.8 ± 0.3 at 20°C (Figure 2B). A similar decline in CO_2_:CH_4_ ratio with temperature was observed in peat incubation experiments from Svalbard in the Arctic (Hoj et al., 2008) as well as in other studies of *Sphagnum*-dominated bogs including S1 bog (Keller and Bridgham, 2007; Ye et al., 2012; Bridgham et al., 2013; Hodgkins et al., 2014; Wilson et al., 2016, 2017). The high CO_2_:CH_4_ ratios appear to be in contradiction with the lack of available inorganic electron acceptors. Thus, based on previous work, we suggest that high CO_2_:CH_4_ ratios in our incubations may reflect the availability of organic electron acceptors in the solid peat (Keller et al., 2009; Keller and Takagi, 2013; Ye et al., 2016; Wilson et al., 2017) and/or the organic matter was simply not mineralized completely and fermentation products may have accumulated. Humic substances as well as a diversity of unsaturated organic compounds have the potential to act as terminal electron acceptors in peatland soils (Lovley et al., 1999; Klupfel et al., 2014; Wilson et al., 2017). The utilization of organic electron acceptors would explain the lag times we observed in CH_4_ production, since CH_4_ is not produced until after the depletion of alternate terminal electron acceptor pools, resulting in a high CO_2_:CH_4_ ratio (Keller et al., 2009; Keller and Takagi, 2013; Ye et al., 2016; Wilson et al., 2017). Furthermore, incomplete mineralization of the organic matter may result in the accumulation of fermentation products and high CO_2_:CH_4_ ratio. In addition, a substantial accumulation of fermentation products was observed at the S1 bog under *in situ* conditions (Zalman et al., 2018) as well as in the Svalbard peat incubation experiments (Tveit et al., 2015), suggesting that terminal processes rather than upstream fermentative steps were rate limiting for CH_4_ production. Although, acetoclastic methanogenesis has been proposed to be a major methane source under low-temperature conditions (Nozhevnikova et al., 2007; Blake et al., 2015), our sequencing efforts failed to recover a substantial population of known acetoclastic methanogens. Our group has studied the *in situ* composition of peat microbial communities at the S1 bog since 2014 by applying a similar next-generation sequencing approach. While deeper sequence efforts coupled with isotopic gas analysis were able to detect the presence of the acetoclastic methanogens in unincubated peat from S1 bog (Lin et al., 2014a,b), such acetoclasts were not observed in lab incubations. Alternatively, we suggest that under low-temperature conditions, hydrogenotrophic methanogens are being outcompeted by other functional guilds such a homoacetogens, which may consume dihydrogen at rates up to 3- to 10-times faster than methanogens, and significantly contribute to total acetate production under psychrophilic conditions (Kotsyurbenko et al., 2001; Ye et al., 2014). On the other hand, low methane maximum potential rates may result from a high concentration of inhibitory compounds produced by the peat moss, *Sphagnum*, which predominates over plant communities at the S1 bog. *Sphagnum* moss releases a wide range of polyphenolic compounds such a sphagnic acids (Rasmussen et al., 1995) with antibiotic and pH-controlling properties (Stalheim et al., 2009) that may directly affect microbial community structure and inhibit methanogens (Medvedeff et al., 2015). Nevertheless, the biochemical properties and thermal stability of these compounds are not yet established.

It is difficult to extrapolate gas production rates from microcosms to the field condition where a host of other processes such as autotrophy, methanotrophy, and aerobic respiration will impact the response of greenhouse gas production/emission to warming. However, initial results from deep peat heating experiments conducted in the field at the S1 bog provide some support for our findings (Wilson et al., 2016): while *in situ* methane fluxes showed a substantial increase with temperature, CO_2_ fluxes measured as dark ecosystem respiration did not, resulting in a decrease in the CO_2_:CH_4_ ratio with warming. Further, the range of CO_2_:CH_4_ ratios from fluxes measured in the field overlaps with that measured in microcosms of S1 bog peat. The consistent decline in CO_2_:CH_4_ ratios with warming, measured in surficial S1 bog peat in the laboratory and in the field (Yvon-Durocher et al., 2014; Wilson et al., 2016), is troubling given that CH_4_ has a sustained global warming potential that is 34-times higher than CO_2_ on a 100-year timescale (Myhre et al., 2014; Neubauer and Megonigal, 2015). Thus, enhanced CH_4_ production due to warming may represent a substantial natural feedback to anthropogenic climate forcing.

### Temperature Response of Microbial Community Diversity and Composition

Whereas microbial abundance showed a direct and positive correlation with activity as measured by CO_2_ and CH_4_ production, a negative correlation was observed between peat microbial diversity and elevated temperature (Figure 4). The diversity of peat microbial communities declined with increasing temperature to 30°C, which is approximately 13°C higher than the average monthly high temperature observed at the S1 bog. Moreover, at temperature treatments above 30°C, no ASV’s affiliated with methanogenesis were detected, suggesting that a threshold was reached above which the microbial communities could not cope with further warming (Sihi et al., 2017) on the timescale of this experiment. Our results are corroborated by field observations of *Sphagnum* microbiomes in a whole ecosystem warming experiment conducted at S1 bog in which a significant reduction of phyllosphere associated microbial and diazotrophic communities was observed (Carrell et al., 2019). Microbial diversity has often been associated with the effective functioning and desirable outcomes for ecosystems (Fierer et al., 2013; Wagg et al., 2014; Delgado-Baquerizo et al., 2016; Kolton et al., 2017; Shade, 2017). Increasing temperatures may impact microbial function and diversity by decreasing carbon usage efficiency due to changes in availability and quality of carbon source, deactivation of enzymes, and exposing microbial communities’ to the higher energy demands required for protein turnover and membrane repair (Fierer et al., 2007; Allison et al., 2010; Chowdhury and Conrad, 2010; Dijkstra et al., 2011; Frey et al., 2013; Okie et al., 2015; Gruner et al., 2017; Sihi et al., 2017). Our results suggest that the exposure of peatland ecosystems to rapid climate change perturbation may lead to a loss in microbial diversity which is likely to impact ecosystem functioning through changes in species composition and/ or functional diversity. However, our observations should be considered as a starting point for further inquiry of ecological mechanisms and community outcomes (Shade, 2017). Further research should elucidate specific aspects of microbial metabolism that are linked to a decline in diversity in response to warming.

In agreement with previous field studies conducted at S1 bog, peat microbial communities were predominated by *Acidobacteria* and *Proteobacteria* (comprising approximately 70% of overall sequence abundance) prior to incubation in this study (Lin et al., 2012, 2014b; Wilson et al., 2016; Zalman et al., 2018). In contrast, pronounced shifts in microbial community composition were observed with warming at the end of the incubation period (Figure 5A). Differences in community composition were characterized by a decrease in the relative abundance of *Acidobacteriia* from 46.7 ± 1% in the unincubated peat samples to 30.7 ± 2 and 1.3 ± 1.3% at the end of the incubation period at 25 and 30°C, respectively (Supplementary Table S6). In contrast, an increase in the relative abundances of *Clostridia* as well as hydrogenotrophic methanogens taxonomically affiliated with the *Methanobacteriaceae* family was observed at all incubation temperatures after end of the experiment relative to the pre-incubation condition. Whereas ours (Figure 5A) and other studies found a small contribution of the *Firmicutes* in unincubated peat microbial communities (Juottonen et al., 2017), their relative abundance rose from 1.3 ± 0.4% in the unincubated peat community to 62.6 ± 2.5% in the community developed by the end of the incubation period at 30°C, and the *Clostridiaceae* family became a dominant member of the post-incubation communities. Members of the *Clostridiaceae* family are spore-forming obligate anaerobes that are well-adapted to survive under extreme conditions found in soils and can utilize a wide range of carbon substrates (Schnurer et al., 1996; Wiegel et al., 2006; Westerholm et al., 2010). Additionally, the relative abundance of taxa capable of fermentation and hydrogen production (*Clostridium, Ethanoligenens* and unclassified members of the family *Ruminococcaceae*) correlated well with CH_4_ maximum potential rates and the succession of *Methanobacterium* (Figure 5C). Moreover, members of the *Clostridiaceae* have been shown to thrive in acidic peat, facilitating fermentation in co-culture with hydrogenotrophic methanogenesis mediated by the *Methanobacteriaceae* (Hunger et al., 2015; Schmidt et al., 2015; Tveit et al., 2015;Juottonen et al., 2017).

Results from anaerobic incubations conducted with peat from the S1 Bog (this study) and Svalbard (Tveit et al., 2015) show that the relative abundance of *Clostridiales* and *Methanobacteriales* are well-correlated with maximum CH_4_ production rates at mesophilic conditions (Supplementary Figure S2). By the end of the incubation period, members of the *Clostridicaeae* family became dominant above the 20°C threshold where overall bacterial abundance was shown to decline. Collectively, these observations suggest that at extreme temperatures well above *in situ* conditions, and in the absence of active photosynthetic organisms, versatile members of the *Clostridiaceae* family will survive and outcompete other bacteria. Surprisingly, only hydrogenotrophic and methylotrophic methanogens were detected during this study (Figure 5B). An analysis of primer coverage against the RDP database^2^ revealed that the 515/806R primer set is biased against *Methanosarcinaceae*, with only 67% of the RDP available sequences detected by these primers (Supplementary Table S8). Members of the *Methanosarcinaceae* utilize a wide range of substrates including carbon mono- and di-oxide, methanol, acetate, butanol, and methylated compounds such as dimethyl-sulfide to produce methane (Oren, 2014). Thus, primer bias may lead to an underrepresentation of this versatile group of acetoclastic methanogens in peat soils of the SPRUCE S1 bog site.

## Conclusion

Climate change models project a temperature increase of 6–10°C for the northern hemisphere by the end of this century. Moreover, it was suggested that the exposure of northern peat to elevated temperatures will have a significant effect on the stability of microbial community composition and activity, leading to alteration of ecosystem functioning. In this study, the temperature response of CO_2_ production under anoxic conditions indicates that separate psychrophilic and mesophilic microbial communities process organic matter in surficial peat from a *Sphagnum*-dominated bog. Methane production was enhanced with increasing incubation temperature while CO_2_ production was diminished, indicating that warming lowers the CO_2_:CH_4_ ratio. Microbial diversity was negatively correlated with temperature, suggesting that prolonged exposure of the peatland ecosystem to elevated temperatures will lead to a loss in microbial diversity. Given the higher warming potential of CH_4_, these observations are concerning and the mechanisms contributing to elevated methane production by archaea at the expense of bacterial CO_2_ production should be further explored.

## Author Contributions

MK conceived of the study. JEK, JPC, and RMW collected samples from the field. MK performed the experiments, the data and statistical analyses and wrote the manuscript. AM carried out the GC measurements. AM, JEK, JPC, and RMW provided valuable insight and ideas during numerous sessions of discussion. All authors provided critical comments on the manuscript and gave final approval for publication.

## Conflict of Interest Statement

The authors declare that the research was conducted in the absence of any commercial or financial relationships that could be construed as a potential conflict of interest.

## Footnotes

1 ncbi.nlm.nih.gov/bioproject

2 https://rdp.cme.msu.edu/

## Supplementary Material

FIGURE S1 Greenhouse gas accumulation in slurries of S1 Bog peat incubated at 13 temperatures. **(A)** Carbon dioxide; **(B)** methane.

FIGURE S2 Relative abundance of *Methanobacteriales* and *Clostridiales*. The relative abundance of the sequences assigned to a given taxonomic level was calculated for each of the biological replicate, and the average value was then used to represent the relative abundance of each temperature treatment. The error bars show the standard deviation of relative abundance for each temperature treatments (*n* = 4).

TABLE S1 Effect of incubation temperature on greenhouse gas production by the S1 Bog peat.

TABLE S2 Effect of incubation temperature on microbial abundance.

TABLE S3 Summary beta diversity statistics based on Bray–Curtis dissimilarity metrics.

TABLE S4 Effect of incubation temperature on microbial alpha diversity.

TABLE S5 Effect of incubation temperature on the relative abundance of the selected Phyla.

TABLE S6 Effect of incubation temperature on the relative abundance of the selected Classes.

TABLE S7 Effect of incubation temperature on the relative abundance of the methanogenic and *Clostridiaceae* communities.

TABLE S8 Primers coverage of the methanogenic members.

## References

[B1] Allison S. D. Wallenstein M. D. Bradford M. A. (2010). Soil-carbon response to warming dependent on microbial physiology. *Nat. Geosci.* 3 336–340. 10.1038/ngeo846

[B2] Angle J. C. Morin T. H. Solden L. M. Narrowe A. B. Smith G. J. Borton M. A. (2017). Methanogenesis in oxygenated soils is a substantial fraction of wetland methane emissions. *Nat. Commun.* 8:1567. 10.1038/s41467-017-01753-4 29146959 PMC5691036

[B3] Bižić-Ionescu M. Klintzch T. Ionescu D. Hindiyeh M. Y. Guenthel M. Muro Pastor A. M. (2018). Widespread formation of methane by Cyanobacteria in aquatic and terrestrial environments. *bioRxiv* [Preprint] 10.1101/398958

[B4] Blake L. I. Tveit A. Øvreås L. Head I. M. Gray N. D. (2015). Response of methanogens in Arctic sediments to temperature and methanogenic substrate availability. *PLoS One* 10:e0129733. 10.1371/journal.pone.0129733 26083466 PMC4471053

[B5] Borrel G. Adam P. S. Gribaldo S. (2016). Methanogenesis and the Wood–Ljungdahl pathway: an ancient, versatile, and fragile association. *Genome Biol. Evol.* 8 1706–1711. 10.1093/gbe/evw114 27189979 PMC4943185

[B6] Borrel G. O’toole P. W. Harris H. M. B. Peyret P. Brugère J.-F. Gribaldo S. (2013). Phylogenomic data support a seventh order of methylotrophic methanogens and provide insights into the evolution of methanogenesis. *Genome Biol. Evol.* 5 1769–1780. 10.1093/gbe/evt128 23985970 PMC3814188

[B7] Bridgham S. D. Cadillo-Quiroz H. Keller J. K. Zhuang Q. (2013). Methane emissions from wetlands: biogeochemical, microbial, and modeling perspectives from local to global scales. *Glob. Change Biol.* 19 1325–1346. 10.1111/gcb.12131 23505021

[B8] Callahan B. J. Mcmurdie P. J. Rosen M. J. Han A. W. Johnson A. J. A. Holmes S. P. (2016). DADA2: high-resolution sample inference from Illumina amplicon data. *Nat. Methods* 13 581–583. 10.1038/nmeth.3869 27214047 PMC4927377

[B9] Canion A. Kostka J. E. Gihring T. M. Huettel M. Van Beusekom J. E. E. Gao H. (2014). Temperature response of denitrification and anammox reveals the adaptation of microbial communities to in situ temperatures in permeable marine sediments that span 50° in latitude. *Biogeosciences* 11 309–320. 10.5194/bg-11-309-2014

[B10] Caporaso J. G. Kuczynski J. Stombaugh J. Bittinger K. Bushman F. D. Costello E. K. (2010). QIIME allows analysis of high-throughput community sequencing data. *Nat. Methods* 7 335–336. 20383131 10.1038/nmeth.f.303 PMC3156573

[B11] Carrell A. A. Kolton M. Warren M. J. Pelletier D. A. Glass J. B. Kostka J. E. (2019). Experimental warming reduces the diversity and functional potential of the *Sphagnum* microbiome. *bioRxiv* [Preprint] 10.1101/194761

[B12] Chowdhury S. P. Conrad R. (2010). Thermal deactivation of high-affinity H2 uptake activity in soils. *Soil Biol. Biochem.* 42 1574–1580. 10.1016/j.soilbio.2010.05.027

[B13] Collins M. Knutti R. Arblaster J. Dufresne J.-L. Fichefet T. Friedlingstein P. (2014). “Long-term climate change: projections, commitments and irreversibility Pages,” in *Climate Change 2013 – The Physical Science Basis: Working Group I Contribution to the Fifth Assessment Report of the Intergovernmental Panel on Climate Change* ed. Intergovernmental Panel on Climate, Change (Cambridge: Cambridge University Press) 1029–1136. 10.1017/cbo9781107415324.024

[B14] Conrad R. (1999). Contribution of hydrogen to methane production and control of hydrogen concentrations in methanogenic soils and sediments. *FEMS Microbiol. Ecol.* 28 193–202. 10.1016/s0168-6496(98)00086-5

[B15] Conrad R. (2009). The global methane cycle: recent advances in understanding the microbial processes involved. *Environ. Microbiol. Rep.* 1 285–292. 10.1111/j.1758-2229.2009.00038.x 23765881

[B16] Davidson E. A. Janssens I. A. (2006). Temperature sensitivity of soil carbon decomposition and feedbacks to climate change. *Nature* 440 165–173. 10.1038/nature04514 16525463

[B17] Delgado-Baquerizo M. Maestre F. T. Reich P. B. Jeffries T. C. Gaitan J. J. Encinar D. (2016). Microbial diversity drives multifunctionality in terrestrial ecosystems. *Nat. Commun.* 7:10541. 10.1038/ncomms10541 26817514 PMC4738359

[B18] Dijkstra P. Thomas S. C. Heinrich P. L. Koch G. W. Schwartz E. Hungate B. A. (2011). Effect of temperature on metabolic activity of intact microbial communities: evidence for altered metabolic pathway activity but not for increased maintenance respiration and reduced carbon use efficiency. *Soil Biol. Biochem.* 43 2023–2031. 10.1016/j.soilbio.2011.05.018

[B19] Dlugokencky E. J. Nisbet E. G. Fisher R. Lowry D. (2011). Global atmospheric methane: budget, changes and dangers. *Philos. Trans. R. Soc. A Math. Phys. Eng. Sci.* 369 2058–2072. 10.1098/rsta.2010.0341 21502176

[B20] Drake H. L. Horn M. A. Wust P. K. (2009). Intermediary ecosystem metabolism as a main driver of methanogenesis in acidic wetland soil. *Environ. Microbiol. Rep.* 1 307–318. 10.1111/j.1758-2229.2009.00050.x 23765883

[B21] Dunfield P. Knowles R. Dumont R. Moore T. R. (1993). Methane production and consumption in temperate and subarctic peat soils: response to temperature and pH. *Soil Biol. Biochem.* 25 321–326. 10.1016/0038-0717(93)90130-4

[B22] Esson K. C. Lin X. Kumaresan D. Chanton J. P. Murrell J. C. Kostka J. E. (2016). *Alpha*- and *Gammaproteobacterial* methanotrophs codominate the active methane-oxidizing communities in an acidic boreal peat bog. *Appl. Environ. Microbiol.* 82 2363–2371. 10.1128/AEM.03640-15 26873322 PMC4959502

[B23] Faith D. P. (1992). Conservation evaluation and phylogenetic diversity. *Biol. Conserv.* 61 1–10. 10.1016/0006-3207(92)91201-3

[B24] Fierer N. Bradford M. A. Jackson R. B. (2007). Toward an ecological classification of soil bacteria. *Ecology* 88 1354–1364. 10.1890/05-1839 17601128

[B25] Fierer N. Ladau J. Clemente J. C. Leff J. W. Owens S. M. Pollard K. S. (2013). Reconstructing the microbial diversity and function of pre-agricultural tallgrass prairie soils in the United States. *Science* 342 621–624. 10.1126/science.1243768 24179225

[B26] Freitag T. E. Prosser J. I. (2009). Correlation of methane production and functional gene transcriptional activity in a peat soil. *Appl. Environ. Microbiol.* 75 6679–6687. 10.1128/AEM.01021-09 19749064 PMC2772438

[B27] Frey S. D. Lee J. Melillo J. M. Six J. (2013). The temperature response of soil microbial efficiency and its feedback to climate. *Nat. Clim. Change* 3 395–398. 10.1111/gcb.14281 29682861

[B28] Glissman K. Chin K. J. Casper P. Conrad R. (2004). Methanogenic pathway and archaeal community structure in the sediment of eutrophic Lake Dagow: effect of temperature. *Microb. Ecol.* 48 389–399. 10.1007/s00248-003-2027-2 15692859

[B29] Gorham E. (1991). Northern peatlands: role in the carbon cycle and probable responses to climatic warming. *Ecol. Appl.* 1 182–195. 10.2307/1941811 27755660

[B30] Gruner D. S. Bracken M. E. S. Berger S. A. Eriksson B. K. Gamfeldt L. Matthiessen B. (2017). Effects of experimental warming on biodiversity depend on ecosystem type and local species composition. *Oikos* 126 8–17. 10.1111/oik.03688

[B31] Hanson P. J. Riggs J. S. Nettles W. R. Phillips J. R. Krassovski M. B. Hook L. A. (2017). Attaining whole-ecosystem warming using air and deep-soil heating methods with an elevated CO2 atmosphere. *Biogeosciences* 14 861–883. 10.5194/bg-14-861-2017

[B32] Hodgkins S. B. Tfaily M. M. Mccalley C. K. Logan T. A. Crill P. M. Saleska S. R. (2014). Changes in peat chemistry associated with permafrost thaw increase greenhouse gas production. *Proc. Natl. Acad. Sci. U.S.A.* 111 5819–5824. 10.1073/pnas.1314641111 24711402 PMC4000816

[B33] Hoj L. Olsen R. A. Torsvik V. L. (2008). Effects of temperature on the diversity and community structure of known methanogenic groups and other archaea in high Arctic peat. *ISME J.* 2 37–48. 10.1038/ismej.2007.84 18180745

[B34] Hunger S. Gossner A. S. Drake H. L. (2015). Anaerobic trophic interactions of contrasting methane-emitting mire soils: processes versus taxa. *FEMS Microbiol. Ecol.* 91:fiv045. 10.1093/femsec/fiv045 25877342

[B35] Juottonen H. Eiler A. Biasi C. Tuittila E. S. Yrjala K. Fritze H. (2017). Distinct anaerobic bacterial consumers of cellobiose-derived carbon in boreal fens with different CO2/CH4 production ratios. *Appl. Environ. Microbiol.* 83:e02533-16. 10.1128/AEM.02533-16 27913414 PMC5288814

[B36] Kahm M. Hasenbrink G. Lichtenberg-Fraté H. Ludwig J. Kschischo M. (2010). grofit: fitting biological growth curves with R. *J. Stat. Softw.* 33 1–21.20808728

[B37] Kaplan W. A. Teal J. M. Valiela I. (1977). Denitrification in salt marsh sediments: evidence for seasonal temperature selection among populations of denitrifiers. *Microb. Ecol.* 3 193–204. 10.1007/BF02010617 24233573

[B38] Keller J. K. Bridgham S. D. (2007). Pathways of anaerobic carbon cycling across an ombrotrophic-minerotrophic peatland gradient. *Limnol. Oceanogr.* 52 96–107. 10.4319/lo.2007.52.1.0096

[B39] Keller J. K. Takagi K. K. (2013). Solid-phase organic matter reduction regulates anaerobic decomposition in bog soil. *Ecosphere* 4 1–12.

[B40] Keller J. K. Weisenhorn P. B. Megonigal J. P. (2009). Humic acids as electron acceptors in wetland decomposition. *Soil Biol. Biochem.* 41 1518–1522. 10.1016/j.soilbio.2009.04.008

[B41] King D. Nedwell D. B. (1984). Changes in the Nitrate-reducing community of an anaerobic saltmarsh sediment in response to seasonal selection by temperature. *Microbiology* 130 2935–2941. 10.1099/00221287-130-11-2935

[B42] Klupfel L. Piepenbrock A. Kappler A. Sander M. (2014). Humic substances as fully regenerable electron acceptors in recurrently anoxic environments. *Nat. Geosci.* 7 195–200. 10.1038/ngeo2084

[B43] Kolton M. Graber E. R. Tsehansky L. Elad Y. Cytryn E. (2017). Biochar-stimulated plant performance is strongly linked to microbial diversity and metabolic potential in the rhizosphere. *New Phytol.* 213 1393–1404. 10.1111/nph.14253 27780299

[B44] Kotsyurbenko O. R. Glagolev M. V. Nozhevnikova A. N. Conrad R. (2001). Competition between homoacetogenic bacteria and methanogenic archaea for hydrogen at low temperature. *FEMS Microbiol. Ecol.* 38 153–159. 10.1016/s0168-6496(01)00179-9

[B45] Lenhart K. Bunge M. Ratering S. Neu T. R. Schüttmann I. Greule M. (2012). Evidence for methane production by saprotrophic fungi. *Nat. Commun.* 3:1046. 10.1038/ncomms2049 22948828

[B46] Limpens J. Berendse F. Blodau C. Canadell J. G. Freeman C. Holden J. (2008). Peatlands and the carbon cycle: from local processes to global implications a synthesis (vol 5, pg 1475, 2008). *Biogeosciences* 5 1739–1739. 10.5194/bg-5-1739-2008

[B47] Lin X. Green S. Tfaily M. M. Prakash O. Konstantinidis K. T. Corbett J. E. (2012). Microbial community structure and activity linked to contrasting biogeochemical gradients in bog and fen environments of the Glacial Lake agassiz peatland. *Appl. Environ. Microbiol.* 78 7023–7031. 10.1128/AEM.01750-12 22843538 PMC3457479

[B48] Lin X. Tfaily M. M. Green S. J. Steinweg J. M. Chanton P. Imvittaya A. (2014a). Microbial metabolic potential for carbon degradation and nutrient (nitrogen and phosphorus) acquisition in an ombrotrophic peatland. *Appl. Environ. Microbiol.* 80 3531–3540. 10.1128/AEM.00206-14 24682299 PMC4018864

[B49] Lin X. Tfaily M. M. Steinweg J. M. Chanton P. Esson K. Yang Z. K. (2014b). Microbial community stratification linked to utilization of carbohydrates and phosphorus limitation in a boreal peatland at Marcell Experimental Forest, Minnesota, USA. *Appl. Environ. Microbiol.* 80 3518–3530. 10.1128/AEM.00205-14 24682300 PMC4018854

[B50] Lovley D. R. Fraga J. L. Coates J. D. Blunt-Harris E. L. (1999). Humics as an electron donor for anaerobic respiration. *Environ. Microbiol.* 1 89–98. 10.1046/j.1462-2920.1999.00009.x 11207721

[B51] Lozupone C. Knight R. (2005). UniFrac: a new phylogenetic method for comparing microbial communities. *Appl. Environ. Microbiol.* 71 8228–8235. 10.1128/aem.71.12.8228-8235.2005 16332807 PMC1317376

[B52] Ma S. Jiang J. Huang Y. Shi Z. Wilson R. M. Ricciuto D. (2017). Data-constrained projections of methane fluxes in a Northern Minnesota Peatland in response to elevated CO2 and warming. *J. Geophys. Res. Biogeosci.* 122 2841–2861. 10.1002/2017jg003932

[B53] Martin M. (2011). Cutadapt removes adapter sequences from high-throughput sequencing reads. *EMBnet J.* 17 10–12.

[B54] McGuire K. L. Treseder K. K. (2010). Microbial communities and their relevance for ecosystem models: decomposition as a case study. *Soil Biol. Biochem.* 42 529–535. 10.1016/j.soilbio.2009.11.016

[B55] McMurdie P. J. Holmes S. (2013). Phyloseq: an R package for reproducible interactive analysis and graphics of microbiome census data. *PLoS One* 8:e61217. 10.1371/journal.pone.0061217 23630581 PMC3632530

[B56] Medvedeff C. A. Bridgham S. D. Pfeifer-Meister L. Keller J. K. (2015). Can *Sphagnum* leachate chemistry explain differences in anaerobic decomposition in peatlands? *Soil Biol. Biochem.* 86 34–41. 10.1016/j.soilbio.2015.03.016

[B57] Metje M. Frenzel P. (2005). Effect of temperature on anaerobic ethanol oxidation and methanogenesis in acidic peat from a northern wetland. *Appl. Environ. Microbiol.* 71 8191–8200. 10.1128/aem.71.12.8191-8200.2005 16332802 PMC1317349

[B58] Metje M. Frenzel P. (2007). Methanogenesis and methanogenic pathways in a peat from subarctic permafrost. *Environ. Microbiol.* 9 954–964. 10.1111/j.1462-2920.2006.01217.x 17359267

[B59] Moonsamy P. V. Williams T. Bonella P. Holcomb C. L. Höglund B. N. Hillman G. (2013). High throughput HLA genotyping using 454 sequencing and the Fluidigm Access Array system for simplified amplicon library preparation. *Tissue Antigens* 81 141–149. 10.1111/tan.12071 23398507

[B60] Muyzer G. De Waal E. C. Uitterlinden A. G. (1993). Profiling of complex microbial populations by denaturing gradient gel electrophoresis analysis of polymerase chain reaction-amplified genes coding for 16S rRNA. *Appl. Environ. Microbiol.* 59 695–700. 7683183 10.1128/aem.59.3.695-700.1993 PMC202176

[B61] Myhre G. Shindell D. Bréon F.-M. Collins W. Fuglestvedt J. Huang J. (2014). “Anthropogenic and natural radiative forcing,” in *Climate Change 2013 – The Physical Science Basis: Working Group I Contribution to the Fifth Assessment Report of the Intergovernmental Panel on Climate Change* ed. Intergovernmental Panel on Climate, Change (Cambridge: Cambridge University Press) 659–740. 10.1017/cbo9781107415324.018

[B62] Neubauer S. C. Megonigal J. P. (2015). Moving beyond global warming potentials to quantify the climatic role of ecosystems. *Ecosystems* 18 1000–1013. 10.1007/s10021-015-9879-4

[B63] Nozhevnikova A. N. Nekrasova V. Ammann A. Zehnder A. J. B. Wehrli B. Holliger C. (2007). Influence of temperature and high acetate concentrations on methanogenesis in lake sediment slurries. *FEMS Microbiol. Ecol.* 62 336–344. 10.1111/j.1574-6941.2007.00389.x 17949433

[B64] Offre P. Spang A. Schleper C. (2013). Archaea in biogeochemical cycles. *Annu. Rev. Microbiol.* 67 437–457. 10.1146/annurev-micro-092412-155614 23808334

[B65] Okie J. G. Van Horn D. J. Storch D. Barrett J. E. Gooseff M. N. Kopsova L. (2015). Niche and metabolic principles explain patterns of diversity and distribution: theory and a case study with soil bacterial communities. *Proc. R. Soc. B Biol. Sci.* 282:20142630. 10.1098/rspb.2014.2630 26019154 PMC4590432

[B66] Oksanen J. Blanchet F. G. Kindt R. Legendre P. Minchin P. R. O’hara R. B. (2015). *Vegan: Community Ecology Package. R package vegan, vers. 2.2–1*.

[B67] Oren A. (2014). “The family methanosarcinaceae,” in *The Prokaryotes: Other Major Lineages of Bacteria and The Archaea* eds Rosenberg E. Delong E. F. Lory S. Stackebrandt E. Thompson F. (Berlin: Springer) 259–281. 10.1007/978-3-642-38954-2_408

[B68] Paulson J. N. Stine O. C. Bravo H. C. Pop M. (2013). Differential abundance analysis for microbial marker-gene surveys. *Nat. Meth.* 10 1200–1202. 10.1038/nmeth.2658 24076764 PMC4010126

[B69] Peltoniemi K. Laiho R. Juottonen H. Bodrossy L. Kell D. K. Minkkinen K. (2016). Responses of methanogenic and methanotrophic communities to warming in varying moisture regimes of two boreal fens. *Soil Biol. Biochem.* 97 144–156. 10.1016/j.soilbio.2016.03.007

[B70] Pietikåinen J. Pettersson M. Bååth E. (2005). Comparison of temperature effects on soil respiration and bacterial and fungal growth rates. *FEMS Microbiol. Ecol.* 52 49–58. 10.1016/j.femsec.2004.10.002 16329892

[B71] Price M. N. Dehal P. S. Arkin A. P. (2010). FastTree 2-approximately maximum-likelihood trees for large alignments. *PLoS One* 5:e9490. 10.1371/journal.pone.0009490 20224823 PMC2835736

[B72] Quast C. Pruesse E. Yilmaz P. Gerken J. Schweer T. Yarza P. (2013). The SILVA ribosomal RNA gene database project: improved data processing and web-based tools. *Nucleic Acids Res.* 41 D590–D596. 10.1093/nar/gks1219 23193283 PMC3531112

[B73] R Development Core Team (2018). *R: A Language and Environment for Statistical Computing*. Viena: R Foundation for Statistical Computing.

[B74] Rasmussen S. Wolff C. Rudolph H. (1995). Compartmentalization of phenolic constituents in *Sphagnum*. *Phytochemistry* 38 35–39. 10.1016/0031-9422(94)00650-i

[B75] Schadel C. Bader M. K. F. Schuur E. A. G. Biasi C. Bracho R. Capek P. (2016). Potential carbon emissions dominated by carbon dioxide from thawed permafrost soils. *Nat. Clim. Change* 6 950–953. 10.1038/nclimate3054

[B76] Schimel J. (2013). Soil carbon: microbes and global carbon. *Nat. Clim. Change* 3 867–868. 10.1038/nclimate2015

[B77] Schloss P. D. Westcott S. L. Ryabin T. Hall J. R. Hartmann M. Hollister E. B. (2009). Introducing mothur: open-source, platform-independent, community-supported software for describing and comparing microbial communities. *Appl. Environ. Microbiol.* 75 7537–7541. 10.1128/AEM.01541-09 19801464 PMC2786419

[B78] Schmidt O. Horn M. A. Kolb S. Drake H. L. (2015). Temperature impacts differentially on the methanogenic food web of cellulose-supplemented peatland soil. *Environ. Microbiol.* 17 720–734. 10.1111/1462-2920.12507 24813682

[B79] Schnurer A. Schink B. Svensson B. H. (1996). *Clostridium ultunense* sp nov, a mesophilic bacterium oxidizing acetate in syntrophic association with a hydrogenotrophic methanogenic bacterium. *Int. J. Syst. Bacteriol.* 46 1145–1152. 10.1099/00207713-46-4-1145 8863449

[B80] Schulz S. Conrad R. (1996). Influence of temperature on pathways to methane production in the permanently cold profundal sediment of Lake Constance. *FEMS Microbiol. Ecol.* 20 1–14. 10.1016/0168-6496(96)00009-8

[B81] Schuur E. A. G. Mcguire A. D. Schadel C. Grosse G. Harden J. W. Hayes D. J. (2015). Climate change and the permafrost carbon feedback. *Nature* 520 171–179. 10.1038/nature14338 25855454

[B82] Sebestyen S. Dorrance C. Olson D. Verry E. Kolka R. Elling A. (2011). “Long-term monitoring sites and trends at the Marcell Experimental Forest,” in *Peatland Biogeochemistry and Watershed Hydrology at the Marcell Experimental Forest* eds Kolka R. Sebestyen S. Verry E. S. Brooks K. (Boca Raton, FL: CRC Press) 15–71. 10.1201/b10708-3

[B83] Shade A. (2017). Diversity is the question, not the answer. *ISME J.* 11 1–6. 10.1038/ismej.2016.118 27636395 PMC5421358

[B84] Sihi D. Inglett P. W. Gerber S. Inglett K. S. (2017). Rate of warming affects temperature sensitivity of anaerobic peat decomposition and greenhouse gas production. *Glob. Change Biol.* 24 e259–e274. 10.1111/gcb.13839 28746792

[B85] Stalheim T. Ballance S. Christensen B. E. Granum P. E. (2009). Sphagnan – a pectin-like polymer isolated from *Sphagnum* moss can inhibit the growth of some typical food spoilage and food poisoning bacteria by lowering the pH. *J. Appl. Microbiol.* 106 967–976. 10.1111/j.1365-2672.2008.04057.x 19187129

[B86] Tarnocai C. Canadell J. G. Schuur E. A. G. Kuhry P. Mazhitova G. Zimov S. (2009). Soil organic carbon pools in the northern circumpolar permafrost region. *Glob. Biogeochem. Cycles* 23:GB2023.

[B87] Tfaily M. M. Cooper W. T. Kostka J. E. Chanton P. R. Schadt C. W. Hanson P. J. (2014). Organic matter transformation in the peat column at Marcell Experimental Forest: humification and vertical stratification. *J. Geophys. Res. Biogeosci.* 119 661–675. 10.1002/2013jg002492

[B88] Thauer R. K. Kaster A.-K. Seedorf H. Buckel W. Hedderich R. (2008). Methanogenic archaea: ecologically relevant differences in energy conservation. *Nat. Rev. Microbiol.* 6 579–591. 10.1038/nrmicro1931 18587410

[B89] Treat C. C. Natali S. M. Ernakovich J. Iversen C. M. Lupascu M. Mcguire A. D. (2015). A pan-Arctic synthesis of CH4 and CO2 production from anoxic soil incubations. *Glob. Change Biol.* 21 2787–2803. 10.1111/gcb.12875 25620695

[B90] Tveit A. T. Urich T. Frenzel P. Svenning M. M. (2015). Metabolic and trophic interactions modulate methane production by Arctic peat microbiota in response to warming. *Proc. Natl. Acad. Sci. U.S.A.* 112 E2507–E2516. 10.1073/pnas.1420797112 25918393 PMC4434766

[B91] Wagg C. Bender S. F. Widmer F. Van Der Heijden M. G. (2014). Soil biodiversity and soil community composition determine ecosystem multifunctionality. *Proc. Natl. Acad. Sci. U.S.A.* 111 5266–5270. 10.1073/pnas.1320054111 24639507 PMC3986181

[B92] Wallenstein M. D. Hall E. K. (2012). A trait-based framework for predicting when and where microbial adaptation to climate change will affect ecosystem functioning. *Biogeochemistry* 109 35–47. 10.1007/s10533-011-9641-8

[B93] Wang Q. Garrity G. M. Tiedje J. M. Cole J. R. (2007). Naïve bayesian classifier for rapid assignment of rRNA sequences into the new bacterial taxonomy. *Appl. Environ. Microbiol.* 73 5261–5267. 10.1128/aem.00062-07 17586664 PMC1950982

[B94] Warren M. J. Lin X. Gaby J. C. Kretz C. B. Kolton M. Morton P. L. (2017). Molybdenum-based diazotrophy in a *Sphagnum* peatland in northern Minnesota. *Appl. Environ. Microbiol.* 83:e01174-17. 10.1128/AEM.01174-17 28667112 PMC5561275

[B95] Weedon J. T. Aerts R. Kowalchuk G. A. Van Logtestijn R. Andringa D. Van Bodegom P. M. (2013). Temperature sensitivity of peatland C and N cycling: does substrate supply play a role? *Soil Biol. Biochem.* 61 109–120. 10.1016/j.soilbio.2013.02.019

[B96] Westerholm M. Roos S. Schnürer A. (2010). *Syntrophaceticus schinkii* gen. nov., sp. nov., an anaerobic, syntrophic acetate-oxidizing bacterium isolated from a mesophilic anaerobic filter. FEMS Microbiol. Lett. 309 100–104. 10.1111/j.1574-6968.2010.02023.x 20546311

[B97] Whitman W. B. Bowen T. L. Boone D. R. (2006). “The methanogenic bacteria,” in *The Prokaryotes: Volume 3: Archaea. Bacteria: Firmicutes, Actinomycetes* eds Dworkin M. Falkow S. Rosenberg E. Schleifer K.-H. Stackebrandt E. (New York, NY: Springer) 165–207.

[B98] Wieder W. R. Bonan G. B. Allison S. D. (2013). Global soil carbon projections are improved by modelling microbial processes. *Nat. Clim. Change* 3 909–912. 10.1038/nclimate1951

[B99] Wiegel J. Tanner R. Rainey F. A. (2006). “An introduction to the family *Clostridiaceae*,” in *The Prokaryotes: Volume 4: Bacteria: Firmicutes, Cyanobacteria* eds Dworkin M. Falkow S. Rosenberg E. Schleifer K.-H. Stackebrandt E. (New York, NY: Springer) 654–678. 10.1007/0-387-30744-3_20

[B100] Wilson R. M. Hopple A. M. Tfaily M. M. Sebestyen S. D. Schadt C. W. Pfeifer-Meister L. (2016). Stability of peatland carbon to rising temperatures. *Nat. Commun.* 7:13723. 10.1038/ncomms13723 27958276 PMC5159855

[B101] Wilson R. M. Tfaily M. M. Rich V. I. Keller J. K. Bridgham S. D. Zalman C. M. (2017). Hydrogenation of organic matter as a terminal electron sink sustains high CO2:CH4 production ratios during anaerobic decomposition. *Org. Geochem.* 112 22–32. 10.1016/j.orggeochem.2017.06.011

[B102] Wu J. Roulet N. T. (2014). Climate change reduces the capacity of northern peatlands to absorb the atmospheric carbon dioxide: the different responses of bogs and fens. *Glob. Biogeochem. Cycles* 28 1005–1024. 10.1002/2014gb004845

[B103] Ye R. Jin Q. Bohannan B. Keller J. K. Bridgham S. D. (2014). Homoacetogenesis: a potentially underappreciated carbon pathway in peatlands. *Soil Biol. Biochem.* 68 385–391. 10.1016/j.soilbio.2013.10.020

[B104] Ye R. Jin Q. Bohannan B. Keller J. K. Mcallister S. A. Bridgham S. D. (2012). pH controls over anaerobic carbon mineralization, the efficiency of methane production, and methanogenic pathways in peatlands across an ombrotrophic–minerotrophic gradient. *Soil Biol. Biochem.* 54 36–47. 10.1016/j.soilbio.2012.05.015

[B105] Ye R. Keller J. K. Jin Q. Bohannan B. J. M. Bridgham S. D. (2016). Peatland types influence the inhibitory effects of a humic substance analog on methane production. *Geoderma* 265 131–140. 10.1016/j.geoderma.2015.11.026

[B106] Yu Y. Lee C. Kim J. Hwang S. (2005). Group-specific primer and probe sets to detect methanogenic communities using quantitative real-time polymerase chain reaction. *Biotechnol. Bioeng.* 89 670–679. 10.1002/bit.20347 15696537

[B107] Yvon-Durocher G. Allen A. P. Bastviken D. Conrad R. Gudasz C. St-Pierre A. (2014). Methane fluxes show consistent temperature dependence across microbial to ecosystem scales. *Nature* 507 488–491. 10.1038/nature13164 24670769

[B108] Yvon-Durocher G. Caffrey J. M. Cescatti A. Dossena M. Giorgio P. D. Gasol J. M. (2012). Reconciling the temperature dependence of respiration across timescales and ecosystem types. *Nature* 487 472–476. 10.1038/nature11205 22722862

[B109] Zalman C. Keller J. K. Tfaily M. Kolton M. Pfeifer-Meister L. Wilson R. M. (2018). Small differences in ombrotrophy control regional-scale variation in methane cycling among Sphagnum-dominated peatlands. *Biogeochemistry* 139 155–177. 10.1007/s10533-018-0460-z

