# Supplementary figures and images for: Impact of Warming on Greenhouse Gas Production and Microbial Diversity in Anoxic Peat From a Sphagnum-Dominated Bog (Grand Rapids, Minnesota, United States)
Source: Front Microbiol. 2019 Apr 26;10:870. doi: 10.3389/fmicb.2019.00870 (PMC6498409; doi:10.3389/fmicb.2019.00870)

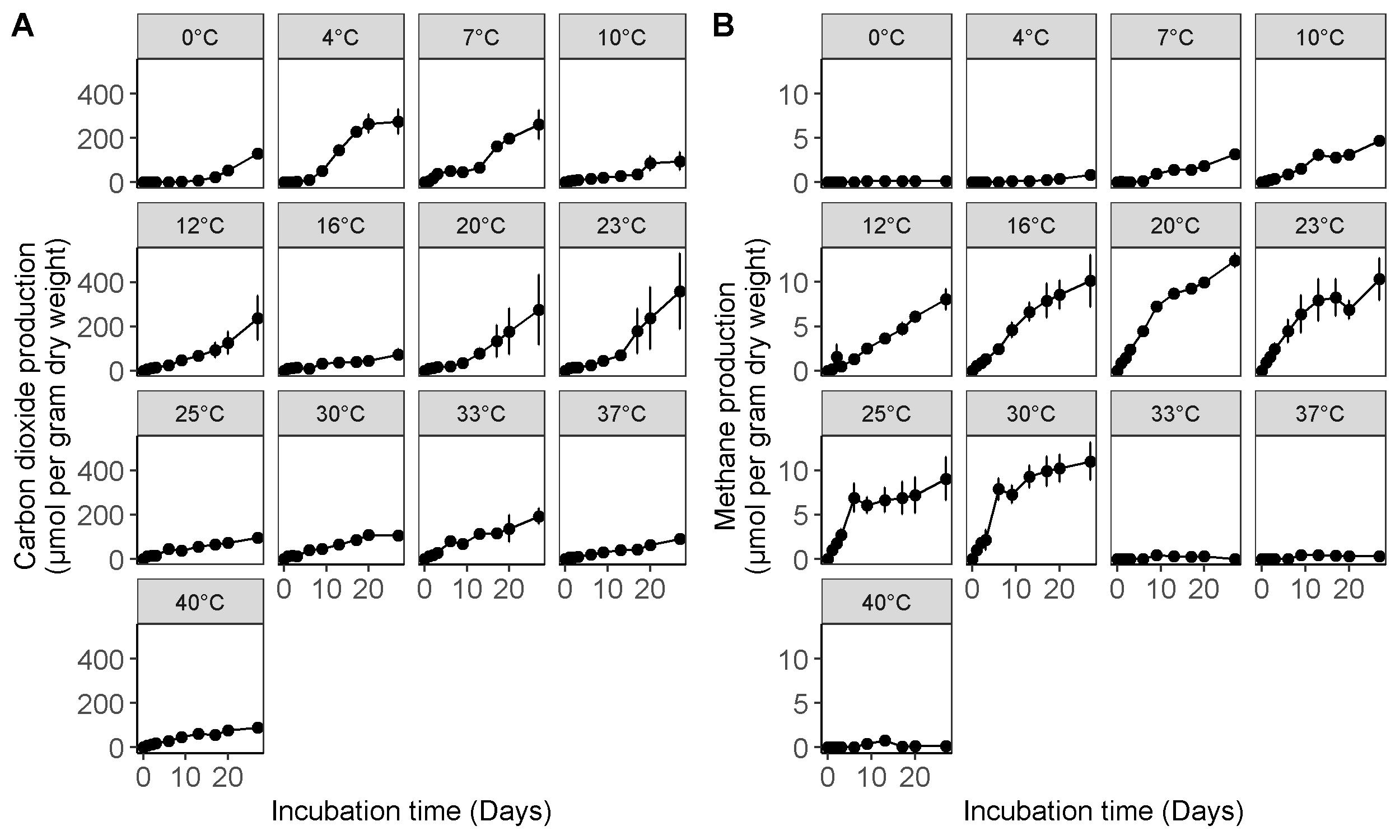

Supplement: FIGURE S1 — Greenhouse gas accumulation in slurries of S1 Bog peat incubated at 13 temperatures. (A) Carbon dioxide; (B) methane. [file Image_1.TIFF]

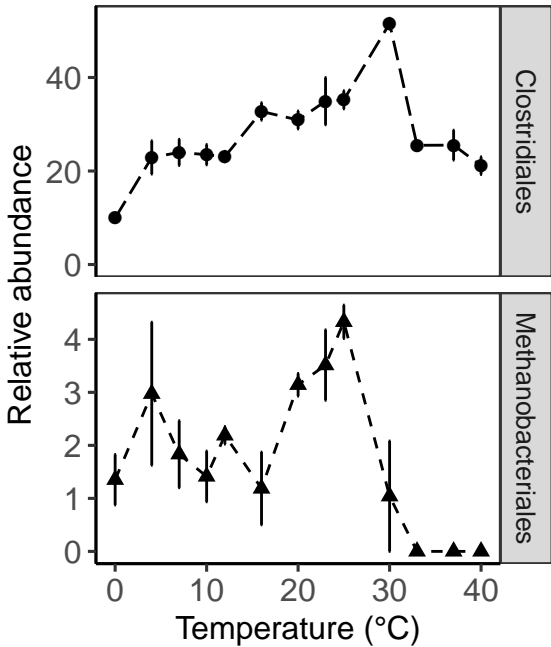

Supplement: FIGURE S2 — Relative abundance of Methanobacteriales and Clostridiales. The relative abundance of the sequences assigned to a given taxonomic level was calculated for each of the biological replicate, and the average value was then used to represent the relative abundance of each temperature treatment. The error bars show the standard deviation of relative abundance for each temperature treatments (n = 4). [file Data_Sheet_1.PDF]
